# When AI speaks like a specialist: ChatGPT-4 in the management of inflammatory bowel disease

**DOI:** 10.3389/frai.2025.1678320

**Published:** 2025-10-10

**Authors:** Elena De Cristofaro, Francesca Zorzi, Maria Abreu, Alice Colella, Giovanna Del Vecchio Blanco, Gionata Fiorino, Elisabetta Lolli, Nurulamin Noor, Loris Riccardo Lopetuso, Mathieu Pioche, Jean Grimaldi, Omero Alessandro Paoluzi, Joana Roseira, Giorgia Sena, Edoardo Troncone, Emma Calabrese, Giovanni Monteleone, Irene Marafini

**Affiliations:** ^1^Gastroenterology Unit, Policlinico Universitario Tor Vergata, Rome, Italy; ^2^Division of Gastroenterology, Department of Medicine, University of Miami Miller School of Medicine, Miami, FL, United States; ^3^Department of Systems Medicine, University of Rome Tor Vergata, Rome, Italy; ^4^Department of Gastroenterology and Digestive Endoscopy, San Camillo-Forlanini Hospital, Rome, Italy; ^5^Department of Gastroenterology, Cambridge University Hospitals NHS Foundation Trust, Cambridge, United Kingdom; ^6^Department of Medicine, University of Cambridge School of Clinical Medicine, Cambridge, United Kingdom; ^7^Medicina interna e Gastroenterologia, CEMAD Centro Malattie dell’Apparato Digerente, Dipartimento di Scienze Mediche e Chirurgiche, Fondazione Policlinico Universitario Gemelli IRCSS, Rome, Italy; ^8^Department of Gastroenterology and Endoscopy, Hôpital Edouard Herriot, Hospices Civils de Lyon, Lyon, France; ^9^Department of Gastroenterology, Unidade Local de Saúde do Algarve, Portimão, Portugal; ^10^ABC - Algarve Biomedical Center, Faro, Portugal

**Keywords:** IBD, artificial inteligence (AI), ulcerative colitis, Crohn, inflammation

## Abstract

**Background:**

Artificial intelligence (AI) is gaining traction in healthcare, especially for patients’ education. Inflammatory bowel diseases (IBD) require continuous engagement, yet the quality of online information accessed by patients is inconsistent. ChatGPT, a generative AI model, has shown promise in medical scenarios, but its role in IBD communication needs further evaluation. The objective of this study was to assess the quality of ChatGPT-4’s responses to common patient questions about IBD, compared to those provided by experienced IBD specialists.

**Methods:**

Twenty-five frequently asked questions were collected during routine IBD outpatient visits and categorized into five themes: pregnancy/breastfeeding, diet, vaccinations, lifestyle, and medical therapy/surgery. Each question was answered by ChatGPT-4 and by two expert gastroenterologists. Responses were anonymized and evaluated by 12 physicians (six IBD experts and six non-experts) using a 5-point Likert scale across four dimensions: accuracy, reliability, comprehensibility, and actionability. Evaluators also attempted to identify whether responses were AI- or human-generated.

**Results:**

ChatGPT-4 responses received significantly higher overall scores than those from human experts (mean 4.28 vs. 4.05; *p* < 0.001). The best-rated scenarios were medical therapy and surgery; the diet scenario consistently received lower scores. Only 33% of AI-generated responses were correctly identified as such, indicating strong similarity to human-written answers. Both expert and non-expert evaluators rated AI responses highly, though IBD specialists gave higher ratings overall.

**Conclusion:**

ChatGPT-4 generated high-quality, clear, and actionable responses to IBD-related patient questions, often outperforming human experts. Its outputs were frequently indistinguishable from those written by physicians, suggesting potential as a supportive tool for patient education. Nonetheless, further studies are needed to assess real-world application and ensure appropriate use in personalized clinical care.

## 1 Introduction

Artificial intelligence (AI) has recently emerged as a powerful tool in healthcare, with applications ranging from diagnostic assistance and image analysis to decision support and patient education (Aung et al., 2021). ChatGPT (Chat Generative Pre-Trained Transformer), developed by OpenAI (San Francisco, CA, USA) and publicly released in November 2022, is an advanced AI language model designed to generate human-like responses based on user input.^1^ Specifically, ChatGPT-4 is a generative language model which, unlike general AI systems, is capable of generating new content by learning patterns from data.

Previous studies in the field of gastroenterology have demonstrated the model’s high level of accuracy in answering scenario-specific medical questions (Maida et al., 2025; Calabrese et al., 2025).

Inflammatory bowel diseases (IBD) are chronic and often disabling conditions that require continuous patient engagement, education, and support (Monteleone et al., 2023; Plevris and Lees, 2022). Patients frequently seek information outside clinical settings, through online resources, social media, and patient communities, to better understand their disease, treatment options, dietary strategies, and general lifestyle advice. However, the quality and reliability of this information are highly variable. A recent study showed that an AI-based system could provide accurate and comprehensive answers to real-world patient questions related to IBD (Sciberras et al., 2024). However, that study used an earlier version of the model (GPT-3), which has notable limitations in terms of language understanding and medical reasoning compared to the more advanced GPT-4, which offers improved contextual comprehension, factual consistency, and clinical relevance, making it a more appropriate tool for assessing the potential role of large language models in healthcare communication (Kung et al., 2023; Harsha Nori et al., 2023). Additionally, the study lacked a direct comparison with responses provided by human experts, introducing a potential bias in the evaluation of accuracy and limiting the validity of its conclusions.

Our study aimed to evaluate the potential role of ChatGPT-4 as a communication tool in IBD care by assessing its responses to a set of commonly asked patient questions and comparing them to those provided by expert gastroenterologists specialized in IBD.

## 2 Methods

### 2.1 Study design and outcomes

In this prospective study, two health professionals, with recognized experience in the field of IBD (IM and FZ), identified the most commonly asked questions by patients with IBD. The list of questions was not arbitrarily created but derived from a one-month observational period in our IBD outpatient clinic, during which the most frequently asked patient questions were systematically recorded. Approximately 500 patients with IBD were seen during this time frame. The 25 most frequently asked questions were selected and categorized into five thematic scenarios, based on a topical review (Supplementary Table 1): 1. Pregnancy and breastfeeding (Questions 1–5); 2. Diet (Questions 6–10); 3. Vaccinations (Questions 11–15); 4. Lifestyle (Questions 16–20); 5. Medical therapy and surgery (Questions 21–25). The complete list of questions and answers is included in Supplementary Table 1.

Each question was independently submitted to ChatGPT-4 using the prompt:

*“If you were a gastroenterologist specialized in inflammatory bowel disease, how would you respond to a patient asking.”*

The same set of questions was also answered by two expert gastroenterologists (IM and FZ) with recognized experience in the management of IBD, who were blinded to the ChatGPT-generated responses. Both generative AI and human experts were instructed simply to “respond to the patient,” without additional constraints on structure or word count. We considered this approach essential to preserve the natural style of each source, reflecting how information would actually be delivered in practice. All responses, whether generated by ChatGPT-4 or by human experts, were randomly assigned to each question and anonymized, ensuring that evaluators were blinded to the source of each response. Subsequently, 12 health professionals were recruited to assess the quality of the responses. This group included six gastroenterologists with expertise in IBD (EC, GF, EL, LRL, NN, JR) and six without specific experience in IBD (GDVB, MP, JG, OAP, GS, ET). IBD experts were defined as physicians with more than 10 years of experience in managing IBD patients, working at a tertiary care centre with dedicated outpatient and endoscopy services, and routinely using advanced therapies. Regarding the “non-expert” group, these were physicians trained in gastroenterology but without a subspecialty focus on IBD (e.g., general gastroenterologists, and endoscopists).

Each participant was asked to evaluate each response using a 5-point Likert scale across four predefined domains: accuracy, reliability, comprehensibility, and actionability (Supplementary Table 2). All evaluators were familiar with the scoring system before starting the assessment. Additionally, participants were asked to indicate, for each response pair, which one they believed had been generated by ChatGPT.

The primary outcome of our study was to evaluate the quality of ChatGPT-4’s responses to a set of commonly asked IBD patient questions, comparing them to those provided by the two gastroenterologist experts in IBD. Secondary outcomes included assessing differences in the evaluation of responses between IBD expert and non-expert clinicians, as well as determining the rate at which evaluators were able to correctly identify ChatGPT-generated responses.

All scores were analysed descriptively using median, mean, interquartile range (IQR), and standard deviation (SD). Comparative analyses were conducted to evaluate differences between AI-generated and human-generated responses, as well as between evaluations provided by IBD experts and non-expert clinicians.

### 2.2 Statistical analysis

Statistical analyses were conducted using SPSS version 29 (IBM Corp., Armonk, NY, USA). Normality of continuous variables was assessed using the Shapiro–Wilk test. As assumptions for parametric testing were not met, only non-parametric tests (Friedman and Mann–Whitney U) were applied. The Friedman test was used to compare paired ordinal scores across multiple items. Differences between IBD experts and non-experts were assessed with the Mann–Whitney U test. A *p*-value < 0.05 was considered statistically significant.

## 3 Results

### 3.1 Comparison between ChatGPT-4 and human responses

**Figure 1:**
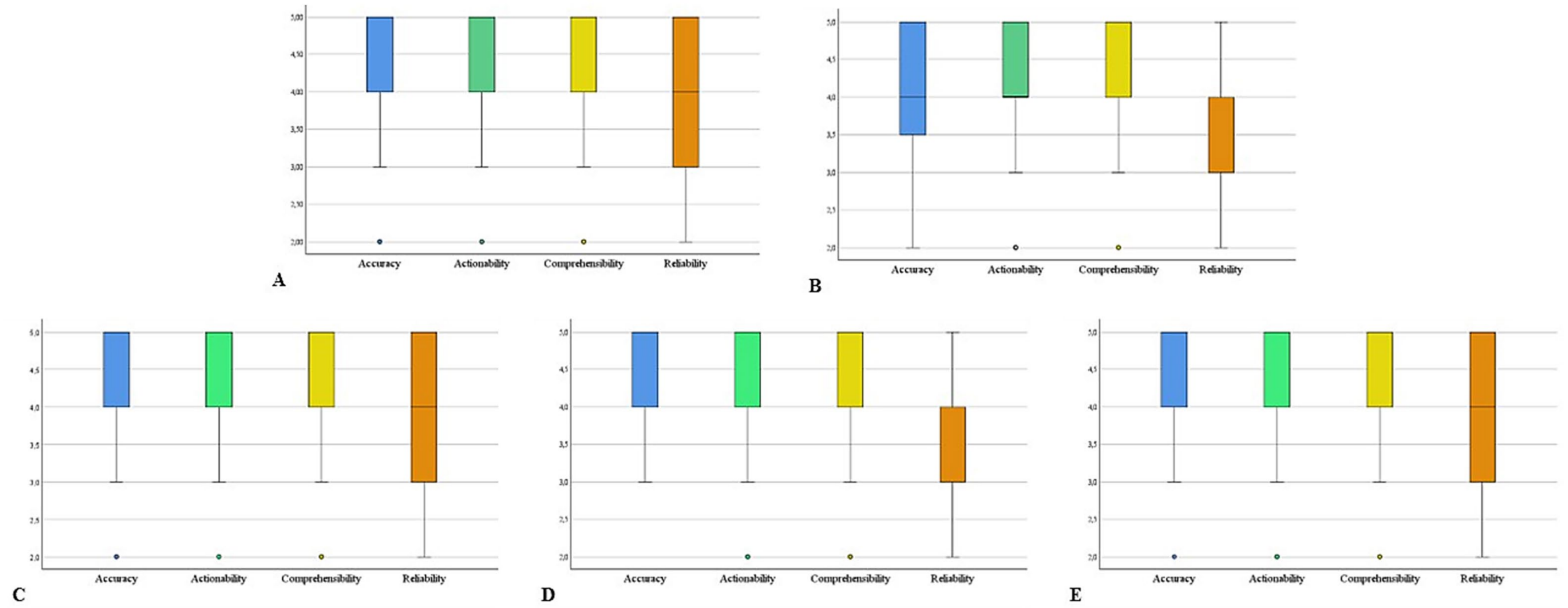
Box plots illustrating the overall ratings of answers generated by humans and ChatGPT across five clinical scenarios: **(A)** pregnancy and breastfeeding, **(B)** diet, **(C)** vaccinations, **(D)** lifestyle, and **(E)** medical therapy and surgery. The complete set of questions and answers for each scenario is provided in Supplementary Table 1.

Among the 12 physicians enrolled in the study, 7 (58%) were male (three in the IBD expert group and four in the non-IBD expert group). The mean age was 42.5 ± 6.0 years in the IBD expert group and 43.5 ± 12.8 years in the non-IBD expert group. Across all questions and evaluators, the average scores were as follows: 4.20 ± 0.76 for accuracy, 3.72 ± 0.96 for reliability, 4.35 ± 0.81 for comprehensibility, and 4.35 ± 0.78 for actionability (Figure 1). Detailed scenario-specific scores are reported in Table 1.

**Table 1:** Detailed schenario-specific scores according to accuracy, reliability, comprehensibility and actionability (5 point Likert scale).

	Pregnancy and breastfeeding (Mean ± SD)	Diet (Mean ± SD)	Vaccinations (Mean ± SD)	Lifestyle (Mean ± SD)	Medical therapy and surgery (Mean ± SD)
Accuracy	4.17 (±0.79)	4.01 (±0.91)	4.12 (±0.64)	4.37 (±0.71)	4.33 (±0.88)
Reliability	3.77 (±0.94)	4.01 (±0.91)	3.80 (±0.90)	3.60 (±0.98)	4.33 (±0.88)
Comprehensibility	4.37 (±0.82)	4.32 (±0.79)	4.31 (±0.83)	4.33 (±0.88)	4.45 (±0.73)
Actionability (5-point Likert scale)	4.37 (±0.77)	4.27 (±0.85)	4.37 (±0.71)	4.33 (±0.88)	4.40 (±0.80)

The highest accuracy was observed in the Medical Therapy and Surgery scenario (mean 4.40 ± 0.71), while the lowest was noted in the Diet scenario (mean 4.01 ± 0.91). Reliability peaked in both the Medical Therapy and Surgery and Vaccinations scenarios (mean 3.87 ± 0.97 and 3.80 ± 0.90, respectively), and was lowest in the Diet and Lifestyle scenarios (mean 3.59 ± 0.99 and 3.80 ± 0.90, respectively). The highest comprehensibility score was recorded in the Medical Therapy and Surgery scenario (mean 4.45 ± 0.73), while the lowest was again found in Vaccinations (mean 4.31 ± 0.83). Actionability was rated highest in the Medical therapy and Surgery scenario (mean 4.40 ± 0.80) and lowest in Diet (mean 4.27 ± 0.85).

A statistically significant difference was observed in the overall evaluation of responses generated by ChatGPT-4 compared to those written by expert gastroenterologists, with ChatGPT responses receiving significantly higher ratings (mean 4.28 ± 0.82 vs. 4.05 ± 0.93; *p* < 0.001). Detailed scores of Chat-GPT4 and human responses for accuracy, reliability, comprehensibility and actionability are reported in Table 2. Regarding the ability to identify the source of responses, only 98 out of 300 ChatGPT-generated answers (33%) were correctly identified by the physicians. Notably, none of the participants correctly identified the ChatGPT-generated response to Question 9 (“Is it helpful to avoid milk and dairy products?”), and only one physician accurately attributed the source of the response to Question 20 (“Does cannabis smoke have a positive effect on IBD?”).

**Table 2:** Scores across metrics (accuracy, reliability, comprehensibility, actionability) for GPT-4 and human responses.

	GPT-4 responses (mean ± SD)	Human responses (mean ± SD)
Accuracy	4.0 (±1.14)	4.0 (±1.14)
Reliability	3.5 (±0.71)	4.0 (±0.0)
Comprehensibility	4.5 (±0.71)	4.5 (±0.71)
Actionability (5-point Likert scale)	4.5 (±0.71)	4.5 (±0.71)

### 3.2 Comparison between IBD experts and non-experts

Both IBD experts and non-expert clinicians assigned high ratings to the responses generated by both humans and Chat-GPT, with experts providing significantly higher scores across all four evaluated dimensions (mean 4.32 ± 0.88 vs. 3.95 ± 0.88; *p* < 0.001). Detailed scores of expert and non-expert-evaluators for accuracy, reliability, comprehensibility and actionability are reported in Table 3. When performance was analysed by thematic scenario, non-experts gave the lowest average ratings in the lifestyle (3.82 ± 0.90) and vaccination (4.11 ± 0.80) scenario, while experts assigned the highest scores in the scenario of medical therapy and surgery (4.57 ± 0.70) and the lowest in the lifestyle scenario (4.23 ± 0.90).

**Table 3:** Scores across metrics (accuracy, reliability, comprehensibility, actionability) for expert and non-expert evaluators (5 point Likert scale).

	Exper evaluators (mean ± SD)	Non-expert evaluators (mean ± SD)
Accuracy	4.44 (±1.14)	3.96 (±0.71)
Reliability	4.05 (±0.71)	3.40 (±0.71)
Comprehensibility	4.50 (±0.71)	4.21 (±0.71)
Actionability	4.52 (±0.71)	4.18 (±0.71)

**Figure 2:**
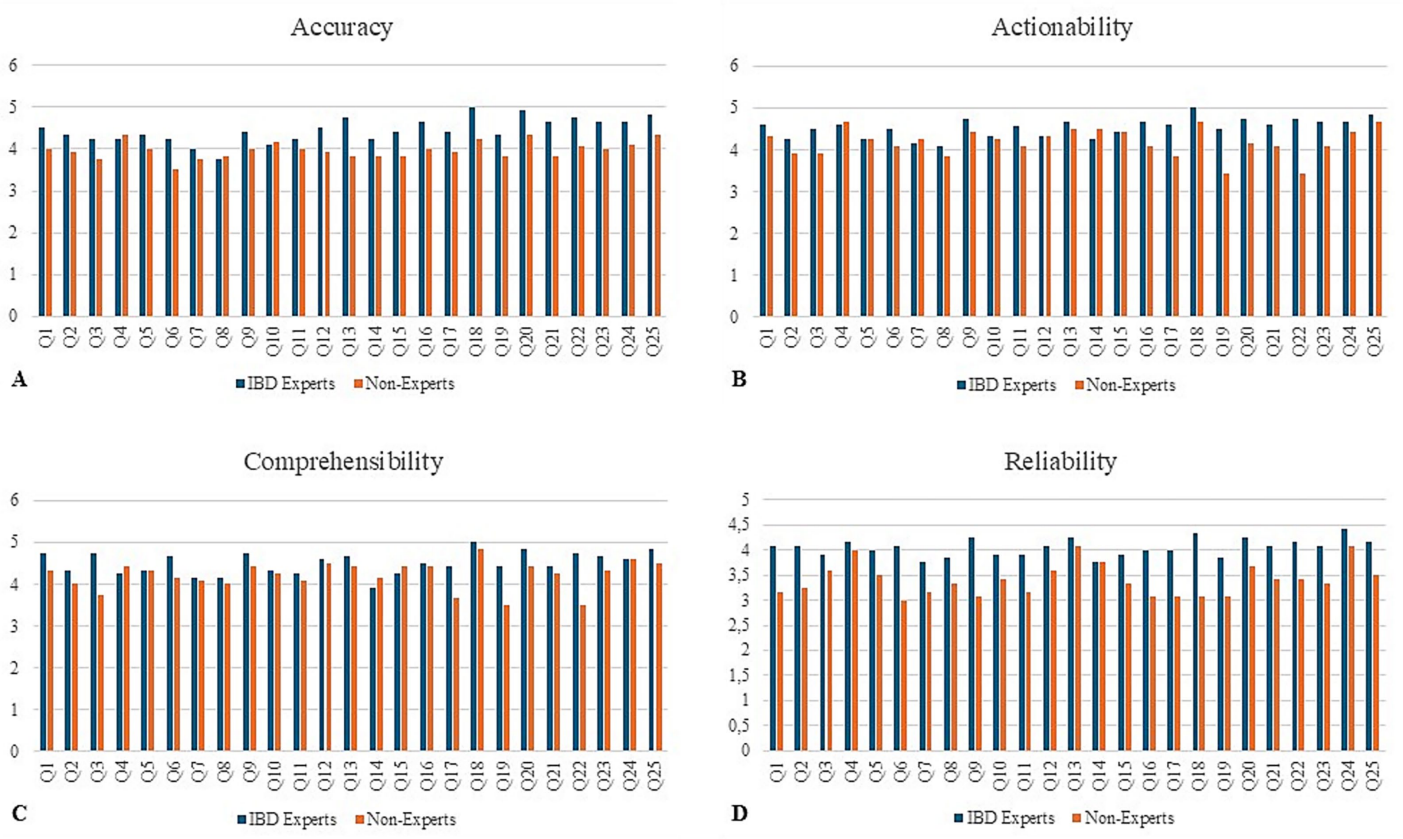
Expert versus non-experts. Mean score per question according to evaluator group (IBD experts vs. non-experts) across the four assessed domains: **(A)** accuracy, **(B)** actionability, **(C)** comprehensibility, and **(D)** reliability. Each bar represents the average rating for one of the 25 questions (Q1–Q25) on a 5-point Likert scale.

Regarding source attribution, 34% of the ChatGPT-generated responses were correctly identified by experts, and 31% were identified by non-experts. Figure 2 provides an overview of the average feature rank values for each of the 25 questions, stratified by physician group.

## 4 Discussion

Our findings confirm the growing potential of generative AI tools in the field of patient education for IBD (Gravina et al., 2024). ChatGPT-4 was able to provide responses that were not only clear and actionable but were often rated higher, particularly in comprehensibility and actionability, than those generated by experienced IBD specialists. These results suggest that generative AI-based language models may have a meaningful role in supplementing traditional physician–patient communication.

Previous work evaluated the performance of ChatGPT-3.5 in answering IBD-related questions based on the European Crohn’s and Colitis Organisation (ECCO) guidelines (Sciberras et al., 2024). While that study demonstrated a generally high level of accuracy, it also reported limited completeness of responses, particularly in complex scenarios such as malignancy screening, vaccination, and family planning. Moreover, the evaluation in that study focused primarily on concordance with clinical guidelines, without including direct comparisons with physician responses or assessments by a diverse panel of clinicians.

Our study builds upon and extends these findings by adopting a more pragmatic and comparative approach. Through the inclusion of both AI-generated and human expert responses, evaluated in a blinded fashion by clinicians with and without IBD-specific expertise, we were able to assess not only the technical quality of the information but also its perceived clarity, reliability, and usefulness in clinical practice. Importantly, ChatGPT-4 responses were frequently rated as superior to those provided by human experts across multiple evaluation scenarios, especially in comprehensibility and actionability. A notable strength of our study is the heterogeneity of evaluators, which allowed us to observe how generative AI responses are perceived by specialists and general gastroenterologists alike. Interestingly, both groups struggled to reliably distinguish between human and AI-generated responses, with only one-third of ChatGPT’s answers correctly attributed. This suggests a high degree of linguistic and stylistic sophistication in the AI’s output, which may enhance its acceptability as a communication aid in clinical settings. While this might appear as “worse than random” performance, it more likely reflects the high similarity between AI and expert responses in both style and content, making discrimination difficult. Interestingly, this systematic inclination to attribute AI outputs to human experts suggests that physicians often perceive ChatGPT-generated answers as indistinguishable from expert-derived ones. Rather than a methodological weakness, we consider this an important finding that highlights the need for further research on source identification, ideally with standardized evaluation frameworks and larger cohorts of evaluators.

A clear discrepancy emerged between IBD experts and non-experts: while experts were generally more critical, emphasizing accuracy and adherence to guidelines, non-experts tended to value clarity and comprehensibility, often assigning higher scores. This divergence reflects the dual importance of technical rigor and communicative accessibility in evaluating patient-directed information.

While ChatGPT-4 performed well across all scenarios, responses in the area of diet consistently received lower ratings, echoing findings from previous studies. This likely reflects the inherent complexity and individual variability of nutritional counselling in IBD, an area where standardized information, no matter how well articulated, cannot fully replace individualized medical advice (Barberio et al., 2025). Consequently, human experts tended to provide cautious and qualified answers, whereas ChatGPT often produced general but less nuanced responses. This contrast may have led evaluators to perceive both sources as less satisfactory compared with other domains. Moreover, many of the evaluators noted that the lack of individualized dietary advice and the frequent reliance on generic statements reduced the perceived accuracy and applicability of responses.

Limitations of our study include the relatively small sample size of physician evaluators and the use of a fixed prompt structure for AI responses, which may not fully capture the dynamic nature of real-life patient interactions. Importantly, the responses were evaluated exclusively by physicians, without input from patients themselves. This may limit the generalizability of our findings, as patients, who often have diverse cultural backgrounds, health literacy levels, and emotional needs, might perceive the clarity, empathy, and usefulness of the responses differently. Indeed, the analysis of patients’ perspective is the focus of an ongoing prospective study at our institution, specifically designed to investigate patient-centered outcomes, using different assessment tools suitable for non health-care professionals. Additionally, the responses were generated by only two experts, which may limit the variability in human answers and may not fully capture the heterogeneity of clinical communication styles. However, it is important to point out that responses were independently assessed by a larger panel of 12 physicians (six IBD and six non-IBD), which helped ensure a balanced evaluation despite the restricted number of experts providing the initial answers.

Finally, although our method enabled standardized comparisons, it does not capture the empathetic and interactive nature of doctor–patient communication, where ChatGPT-4’s clarity and neutrality, though potentially enhancing perceived reliability, cannot replace the nuanced judgment, contextual awareness, and personalization intrinsic to human clinicians.

In conclusion, our results support the idea that ChatGPT-4 can serve as a valuable supplementary tool in patient education for IBD. Its ability to generate clear, high-quality responses that are often indistinguishable from those of medical experts opens new possibilities for enhancing digital health communication. Further research is needed to explore how such tools can be safely and effectively integrated into clinical practice, ensuring both accuracy and patient trust.

## Data Availability

The raw data supporting the conclusions of this article will be made available by the authors, without undue reservation.

## Footnotes

^1^ https://chat.openai.com
