# Supplementary material for: When AI speaks like a specialist: ChatGPT-4 in the management of inflammatory bowel disease
Source: Front Artif Intell. 2025 Oct 10;8:1678320. doi: 10.3389/frai.2025.1678320 (PMC12549657; doi:10.3389/frai.2025.1678320)
Supplement: Supplementary file 1 [file Data_Sheet_1.DOCX]

**Supplementary Table 1.** Questions posed to ChatGPT-4 and to human experts, along with their respective answers presented in random order as shown to the evaluators.

**PREGNANCY AND BREASTFEEDING**

1. **Do chronic inflammatory bowel diseases get worse during pregnancy?**
2. The impact of pregnancy on the clinical course of inflammatory bowel disease varies depending on the specific diagnosis. Women with ulcerative colitis are more likely to experience flare-ups during pregnancy and after childbirth compared to those who are not pregnant. In contrast, pregnancy generally has little to no effect on the course of Crohn’s disease, and in some cases, it may even have a beneficial effect. However, in both ulcerative colitis and Crohn’s disease, conceiving while the disease is active can negatively affect the disease course during pregnancy and increases the risk of relapse and pregnancy related complications. For this reason, it’s recommended to plan pregnancy during a period of clinical remission.
3. Chronic inflammatory bowel diseases, like Crohn’s disease and ulcerative colitis, do not usually worsen during pregnancy, especially if you conceive while the disease is in remission. In fact, being in remission at conception is the best predictor of a stable course throughout pregnancy. However, if the disease is active at the time of conception, there’s a higher chance it may stay active or flare during pregnancy. That’s why it’s important to plan pregnancy during a stable phase and continue appropriate treatment under medical supervision
4. **Can I continue biological therapy with anti-TNFs during pregnancy and breastfeeding?**
5. Yes, anti-TNF therapies (such as infliximab and adalimumab) can be continued during pregnancy, especially in the first two trimesters, to maintain disease remission, which is crucial for a healthy pregnancy. If needed, therapy can also be continued into the third trimester, though some experts suggest spacing the last dose to reduce fetal exposure. During breastfeeding, anti-TNFs are considered safe: they pass into breast milk in minimal, non-active amounts and do not harm the infant. Therefore, both pregnancy and breastfeeding are compatible with continued anti-TNF treatment.
6. Yes. Most medications used to treat inflammatory bowel disease are considered low risk during pregnancy and breastfeeding. Among these, anti-TNF agents stand out, as they have the most extensive long-term safety data among advanced therapies. As a result, anti-TNFs are generally regarded as safe for use during both pregnancy and lactation. It is recommended to continue treatment through the third trimester and to resume it as soon as possible after delivery. If the disease is not well controlled in the third trimester, anti-TNF therapy can be continued right up until delivery and restarted promptly afterward. Overall, anti-TNF treatment is considered low risk and should be maintained during breastfeeding.
7. **Can I continue biological therapy with vedolizumab, ustekinumab and anti-IL-23 during pregnancy and lactation?**
8. Yes, vedolizumab and ustekinumab can be continued during pregnancy if clinically indicated, especially if they are maintaining remission. Although long-term safety data are still growing, no significant increase in adverse pregnancy outcomes has been observed. Anti-IL-23 agents (like risankizumab) have more limited data, but preliminary evidence is reassuring. During breastfeeding, these drugs are considered likely safe, as monoclonal antibodies are poorly absorbed by the infant’s gut. Therefore, their continuation may be justified when benefits outweigh risks, always with close medical monitoring.
9. Yes. Data on the use of vedolizumab and ustekinumab during pregnancy in IBD patients are limited. However, based on current evidence, both drugs are considered low risk and can be continued during pregnancy, usually up to the third trimester. Similarly, although data during breastfeeding are also limited, vedolizumab and ustekinumab are currently regarded as low-risk treatments and can be continued while breastfeeding. In IBD, data on the use of ani-IL-23 during pregnancy and breastfeeding are scarce. However, based on data from ustekinumab, the use of anti-IL-23 agents is generally considered low risk during pregnancy and lactation by most physicians. However, this needs to be confirmed by future studies.
10. **Can I continue therapy with JAK-inhibitors during pregnancy and lactation?**
11. No. JAK inhibitors have been shown to cause serious malformations in animal studies, and there are currently no safety data available in humans. As a result, treatment with any JAK inhibitor is considered contraindicated during pregnancy and breastfeeding.
12. No, JAK-inhibitors (like tofacitinib or upadacitinib) are not recommended during pregnancy or breastfeeding. Animal studies have shown potential risks to the fetus, and human data are still very limited. For this reason, guidelines advise stopping JAK-inhibitors before conception, with an appropriate washout period. These drugs also pass into breast milk and could affect the baby, so they should be avoided during lactation as well. If you’re planning pregnancy or already pregnant, we should discuss switching to a safer treatment option for you and your baby.
13. **Can I do vaginal delivery if I have IBD?**
14. Yes. Vaginal delivery remains the preferred mode of childbirth in patients with IBD, and there is no evidence suggesting worse outcomes with vaginal delivery in cases of inactive perianal disease. However, in certain situations, such as active perianal disease, a history of rectovaginal fistula, or following restorative proctocolectomy, a cesarean section is recommended. This decision should be made after a multidisciplinary discussion involving gastroenterologists, obstetricians, IBD surgeons and the patient.
15. Yes, vaginal delivery is generally safe for most patients with IBD, especially if the disease is in remission and there are no active perianal lesions. The choice between vaginal delivery and cesarean section should follow standard obstetric criteria. However, in patients with active perianal Crohn’s disease or history of complex perianal fistulas or severe anorectal surgery, cesarean section is preferred to avoid perineal trauma. A multidisciplinary evaluation with your obstetrician and gastroenterologist is recommended to plan the safest delivery approach for you and your baby.

**DIET**

1. **What can I eat?**
2. Diet has a profound impact on overall health, and this is especially true for patients with IBD, as it influences the intestinal microbiota, epithelial cells, and immune cells, factors that play a key role in the pathogenesis and perpetuation of inflammation in IBD. Despite this, there is no one-size-fits-all diet for patients with IBD. Most of the evidence supporting these effects comes from preclinical studies, and much of this data still needs to be confirmed in humans. Preclinical evidence shows that a diet high in fat and salt, and low in fiber, promotes intestinal inflammation. Food processing also contributes to gut dysbiosis and inflammation. Additionally, the consumption of food additives may negatively affect gut homeostasis. Consuming a well-balanced diet made primarily from fresh ingredients, while avoiding emulsifiers, additives, and processed foods, is likely beneficial and non-harmful for most IBD patients.
3. There is no universal “IBD diet.” In remission, a balanced diet including fruits, vegetables, grains, proteins, and healthy fats is encouraged. During active flares, it may help to reduce irritating foods such as fatty meals, spicy foods, alcohol, and insoluble fibers. The key is to tailor the diet to your personal tolerance and avoid unnecessary restrictions that may lead to nutritional deficiencies. Dietitian support is highly recommended.
4. **Can I eat fibers?**
5. Yes, especially in remission. Fiber is not harmful and may even be protective. Soluble fiber (found in oats, bananas, potatoes) is usually well tolerated and beneficial. However, during active disease—particularly if you have strictures or narrowing—it may be necessary to temporarily reduce fiber intake to avoid symptoms like bloating or obstruction.
6. Yes. Fibers have a beneficial effect on intestinal homeostasis in both healthy individuals and those with IBD. Therefore, they should be included in the daily diet of patients whose disease is in clinical remission. However, in cases of active disease, particularly when accompanied by diarrhea, fibers intake may need temporarily to be reduced. The main exception of this general advice is patients with Crohn’s disease who have a confirmed intestinal stricture — in these cases, fibers should be avoided as it may increase the risk of developing an occlusive condition.
7. **Can I drink coffee?**
8. Coffee is not contraindicated, but it may increase symptoms such as urgency or diarrhea in some patients. If you tolerate it well, moderate intake is acceptable. If it triggers symptoms, especially during active disease, it’s best to limit or avoid it.
9. Yes. When it comes to coffee consumption, the same guidelines that apply to the general population also apply to individuals with IBD, as there is no evidence that caffeinated beverages worsen the course of the disease. In fact, although limited, some data suggest that caffeine may have a protective effect in patients with ulcerative colitis.
10. **Is it helpful to avoid milk and dairy products?**
11. No. There is no clear evidence that milk or dairy products affect the incidence or progression of IBD. As such, these foods should not be excluded from the diet unless there is a confirmed coexisting condition, such as lactose intolerance.
12. Only if you have lactose intolerance or notice symptoms after consuming dairy. Removing dairy without a clear reason is not advised, as it can reduce your intake of calcium and vitamin D—nutrients that are important in IBD, especially if you are on corticosteroids or have risk of bone loss.
13. **Is it helpful to remove gluten from the diet?**
14. Not unless you have confirmed celiac disease. There is no evidence that a gluten-free diet improves IBD outcomes. Unnecessary exclusion of gluten can lead to a more restrictive and less balanced diet, so it should only be considered under medical or dietetic supervision.
15. No. There is no clear evidence that gluten affect the incidence or progression of IBD. As such, gluten containing-food should not be excluded from the diet unless there is a confirmed coexisting condition, such as celiac disease or gluten sensitivity.

**VACCINES**

1. **Can I get vaccines if I have IBD?**
2. Yes. Vaccines can be safely administered to patients with IBD, and most are actually recommended for this group, ideally at the time of diagnosis and annually thereafter, such as the influenza vaccine. The main exception involves live-virus vaccines, which should be avoided during immunosuppressive therapy and replaced with inactivated alternatives. There is no evidence to suggest that vaccines trigger disease reactivation in individuals with IBD.
3. Yes, patients with IBD can and should receive most vaccines. Inactivated (non-live) vaccines, such as influenza, COVID-19, pneumococcal, and HPV, are safe and effective even during immunosuppressive therapy. Live vaccines (e.g., MMR, varicella) are generally contraindicated if you are on immunosuppressants or biologics, but may be given before starting such treatments. It’s important to stay up to date with vaccinations to reduce infection risk, especially if you are on long-term immunosuppression.
4. Can I get vaccines if I am doing biological therapy?
5. Yes. All non-live (inactivated) vaccines can be safely administered during biologic therapy. In contrast, live vaccines are not considered safe for patients receiving biologic treatment and should ideally be given 1 to 6 months prior to the initiation of immunosuppressive therapy. If this is not feasible, immunosuppressive treatment should be paused and restarted after live vaccines administration after an appropriate safety interval, typically at least 4 weeks.
6. Yes, patients with IBD can and should receive most vaccines, as recommended by ECCO. Inactivated (non-live) vaccines—such as influenza, COVID-19, pneumococcal, and HPV—are safe and effective even during immunosuppressive therapy. Live vaccines (e.g., MMR, varicella) are generally contraindicated if you are on immunosuppressants or biologics, but may be given before starting such treatments. It’s important to stay up to date with vaccinations to reduce infection risk, especially if you are on long-term immunosuppression. Your gastroenterologist or primary care provider should coordinate vaccination planning with you.
7. Do I need to get vaccines before starting biological therapy?
8. Yes. Your vaccination history should ideally be reviewed at the time of diagnosis, or at the very least before starting any biological or immunosuppressive treatment. Any missing vaccinations should be given before therapy begins. If it's clinically safe to delay starting treatment, live vaccines should be considered beforehand in line with your country’s health guidelines. Specifically, live vaccines need to be administered at least 4 weeks before beginning therapy and at least 1 to 6 months after treatment ends (1 month if high-dose steroids were used) before receiving any further live vaccines.
9. Yes, it’s strongly recommended to update your vaccination status before starting biological therapy. This is the safest time to administer live vaccines (like MMR or varicella), which are generally contraindicated once immunosuppression begins. Inactivated vaccines (e.g., flu, COVID-19, hepatitis B) can be given before or after starting treatment, but giving them in advance may lead to a better immune response. A pre-treatment vaccination review helps protect you from infections during therapy and should be part of your care plan.
10. Should I get the flu vaccine every year if I have IBD?
11. Yes, you should receive the flu vaccine every year if you have IBD, regardless of whether you're in remission or on treatment. ECCO and national guidelines recommend annual influenza vaccination because patients with IBD—especially those on immunosuppressants or biologics—have a higher risk of complications from the flu. The vaccine is inactivated and safe, even during flare-ups or while on therapy. It helps prevent serious illness and reduces hospitalizations, making it a key part of preventive care in IBD.
12. Yes. Annual flu shots are recommended for people with IBD, especially those on immunosuppressive therapy, as they are at greater risk for severe influenza. National guidelines recommend annual vaccination, but live flu vaccines should be avoided in patients who are immunosuppressed.
13. Are live vaccines safe for people with IBD?
14. It depends. Live vaccines usually trigger a stronger immune response than inactivated ones. However, they are generally **not safe** for people with IBD who are on immunosuppressive therapy or have significant malnutrition. If needed, live vaccines should be given at least **4 weeks before** starting treatment or **1 to 6 months after** stopping it (1 month for high-dose steroids). The decision should always be made on a case-by-case basis.
15. Live vaccines (such as MMR, varicella, and yellow fever) are generally safe for people with IBD only if they are not on immunosuppressive therapy. If you are taking corticosteroids, immunomodulators (like azathioprine), or biologics, live vaccines are contraindicated due to the risk of infection. If a live vaccine is needed, it should be given before starting immunosuppressive treatment, ideally at least 3–4 weeks in advance. In all cases, vaccination planning should be done with your IBD specialist to balance safety and protection.

**LIFESTYLE**

1. Can I get a tattoo while on biological therapy?
2. Yes, you can get a tattoo, but it’s important to be cautious. Make sure to choose a licensed tattoo studio that follows proper health and hygiene standards. People with IBD taking TNFα inhibitors may have a slightly higher risk of melanoma, so it's a good idea to have your skin checked before getting a tattoo to avoid inking over suspicious lesions. Some people with IBD also have skin conditions like psoriasis. Although psoriasis isn’t a strict contraindication for tattoos, getting tattooed during an active flare-up might trigger a skin reaction known as the Koebner phenomenon, where new psoriasis lesions develop at the site of skin trauma. Tattooing may also trigger a general psoriasis flare. There’s also a small risk of other complications like infection or allergic reactions, risks that are slightly higher when you're on immunosuppressive therapy.
3. Yes, you can get a tattoo while on biological therapy, but extra precautions are necessary. Being on biologics may slightly increase your risk of infection and delayed healing, so it's important to choose a licensed, hygienic tattoo studio that follows strict aseptic techniques. Avoid getting tattooed during disease flares or if you're on high-dose corticosteroids or combination immunosuppression. Aftercare is essential—keep the area clean and monitor for signs of infection.
4. Can I drink alcohol if I have IBD?
5. Yes, you can drink alcohol in moderation if you have IBD and are in remission, but it’s important to be cautious. Alcohol does not directly cause flares, but it can irritate the gut and worsen symptoms like diarrhea or abdominal pain, especially during active disease. It may also interact with some medications (like methotrexate or thiopurines) and increase liver toxicity. Occasional light drinking (e.g., a glass of wine or beer) is generally acceptable if well tolerated, but regular or heavy use is discouraged. Always discuss alcohol use with your IBD team.
6. Yes, in moderation. There's limited scientific evidence, but many doctors suggest drinking alcohol in line with general population guidelines. While red wine may have some anti-inflammatory effects, alcohol can also trigger relapses or worsen GI symptoms in some people with IBD. It can also interfere with how certain medications work or increase side effects. Talk to your doctor if you’re unsure how alcohol might affect you.
7. Can I carry the syringes of biological therapy on the plane?
8. Yes, you can travel with your medication. Use a small cooler bag with ice packs to keep your doses at the correct temperature. Always carry your medication in your hand luggage, never check it in. Declare it at airport security, and bring a doctor’s note and your prescription. If possible, keep medications in their original packaging.
9. Yes, you can carry your biological therapy syringes or pens on the plane. It’s important to keep them in your hand luggage, not in checked baggage, to avoid temperature changes or damage. Make sure to bring a medical certificate or prescription stating you need the medication and needles—this helps with airport security. If the drug requires refrigeration, use a cool bag with ice packs. Also, keep the medication in its original packaging and inform security staff if needed. Many patients travel this way without any issues.
10. Can I have anal and vaginal sex if I have IBD?
11. Yes, both anal and vaginal sex are generally safe if you have IBD, especially when the disease is in remission. Vaginal sex is not typically affected unless there is significant abdominal discomfort or pelvic surgery. Anal sex requires more caution, particularly in Crohn’s disease with a history of perianal disease, fissures, or fistulas, as it may cause pain or worsen local inflammation. Communication with your partner and listening to your body is important. If you experience pain, bleeding, or fear of injury, talk to your gastroenterologist or a pelvic floor specialist for guidance.
12. Yes. However, some people with IBD may have inflammation, abscesses, or fistulas in the genital or anal area, which can make sex uncomfortable or painful. If you’re experiencing these symptoms, it’s worth talking to your doctor for advice.
13. Does cannabis smoke have a positive effect on IBD?
14. No, not reliably. While cannabis may help some people with symptom relief (like pain or nausea), it hasn’t been shown to reduce inflammation or promote healing in the gut. Additionally, regular use can come with risks like dependency, inconsistent dosing, and cannabinoid hyperemesis syndrome (a condition causing severe nausea and vomiting).
15. Some patients with IBD report that cannabis helps relieve symptoms like pain, nausea, or poor appetite, but according to current evidence and guidelines, cannabis does not reduce inflammation or induce remission. So while it might make you feel better temporarily, it doesn’t treat the disease itself. Smoking cannabis can also have side effects—on memory, mood, and lung health—and may interact with other medications. For now, it’s not recommended as a treatment for IBD, but research is ongoing. Always talk to your doctor before considering it.

**MEDICAL THERAPY AND SURGERY**

1. Can I take biological therapy if I have a fever?
2. If you have a fever, it’s generally advised to delay your biological therapy until the cause is clarified. A fever may indicate an infection, and continuing biologics during an active infection can increase the risk of complications. Therapy should be postponed in the presence of systemic infections or signs of fever until resolved or properly evaluated. Always inform your doctor—minor viral illnesses (like a cold) may not require stopping therapy, but bacterial infections, especially with high fever, do. A clinical assessment is essential before continuing treatment.
3. No. A fever could signal an infection, and taking biologics during an active infection is not advised. Contact your doctor first to rule out any underlying infections. You can restart your therapy once the infection has resolved.
4. Does biological therapy increase the risk of cancer?
5. Biological therapy, particularly anti-TNF agents, does not significantly increase the overall risk of cancer in patients with IBD. Some studies have noted a slightly increased risk of certain cancers, such as non-melanoma skin cancers or lymphomas, especially when biologics are combined with immunomodulators (like azathioprine). However, the absolute risk remains low, and the benefit of controlling inflammation outweighs the potential risk. Regular skin checks and age-appropriate cancer screening are recommended as part of routine IBD care.
6. Not overall. It’s difficult to separate the risks from the medication and the disease itself, especially since many patients try multiple therapies over time. That said, some treatments may slightly increase the risk of specific cancers.

- TNFα-antagonists alone do not increase the overall risk, but may raise the chance of lymphoma and melanoma.
- Combination therapy with TNFα-antagonists and thiopurines carries a higher lymphoma risk than either alone, especially for young men under 30 with Crohn’s disease.
- Hepatosplenic T-cell lymphoma, a rare cancer, has been linked to this combo therapy in younger males.
- For vedolizumab, JAK inhibitors, anti-IL-12/23, and anti-IL-23 therapies, long-term cancer risk data is still limited, but no major concerns have been reported so far.

1. Can I get pregnant if I have an ostomy?
2. Yes. Many people with an ostomy go on to have healthy pregnancies and babies. With close monitoring from a multidisciplinary medical team (including gastroenterologists, colorectal surgeons, and maternal-fetal specialists), most pregnancies progress well. As your belly grows, your stoma and the skin around it might change, which could require adjustments to your ostomy equipment, especially during the third trimester.
3. Yes, many women with an ostomy due to IBD can have a safe and successful pregnancy. An ostomy is not a contraindication to pregnancy, and most women can carry to term and deliver safely. However, it's important to have preconception counseling and to be followed by a multidisciplinary team (gastroenterologist, obstetrician, and surgeon if needed). The ostomy may change in size or function during pregnancy, but most issues can be managed. Vaginal delivery is often possible, unless there are other surgical or obstetric indications for cesarean section.
4. How many days should I stay in the hospital for ileo-caecal resection surgery?
5. Most patients stay between 3 and 7 days. Planned surgeries usually mean shorter hospital stays compared to emergency ones. Recovery time may vary based on the extent of the surgery, whether it was done laparoscopically or through open surgery, your nutritional status, and any complications.
6. For most patients undergoing ileo-caecal resection, the hospital stay is typically around 5 to 7 days, especially if the surgery is done laparoscopically. Thanks to modern recovery protocols, like ERAS (Enhanced Recovery After Surgery), we encourage early mobilization and feeding to speed up recovery. If there are no complications—such as infection, delayed bowel function, or pain control issues—you may even go home sooner. Of course, the length of stay can vary based on your overall health and how your body responds after surgery.
7. Can I swim with an ostomy?
8. Yes, absolutely! You can swim with your pouch on, it’s water-resistant and designed to stay sealed. Here are some tips:

- Check your seal before getting in the water.
- Empty your pouch beforehand and try to swim at a time when you're less likely to have output.
- Wait at least an hour after applying your wafer before swimming.
- If your pouch has a filter, use a filter cover sticker to keep water out. Remove it afterward.
- Wear whatever makes you feel comfortable, whether it’s a swimsuit, tankini, or swim trunks.
- Bring extra supplies just in case.
- For added peace of mind during long swims, consider using barrier strips.

For more info and support, check out [www.ostomy.org/swimming-with-an-ostomy](http://www.ostomy.org/swimming-with-an-ostomy).

1. Yes, you can absolutely swim with an ostomy. Having a stoma does not prevent you from enjoying the sea or the pool. Modern ostomy bags are waterproof and adhesive, and many patients swim regularly without issues. You can use additional support like stoma guards, waterproof tapes, or ostomy belts for extra security if needed. It’s best to empty the bag before entering the water, and some prefer smaller or more discreet bags for comfort. Swimming is also a great form of physical activity in IBD and is encouraged if you feel well.

**Supplemetary table 2:** Likert scales for assessment of Accuracy, Actionability, Comprehensibility and Reliability

**Accuracy rating by a 5 point Likert scale**

1. Completely incorrect
2. More incorrect than correct
3. Approximately equally correct and incorrect
4. More correct than incorrect
5. Completely correct

**Actionability rating by a 5-point Likert scale**

1. Not actionable: provides no clear steps or recommendations
2. Minimally actionable: provides vague or incomplete suggestions
3. Moderately actionable: provides basic steps that could be followed
4. Mostly actionable: provides clear steps with minor limitations
5. Highly actionable: provides clear, specific, and practical recommendations that can be easily followed

**Comprehensibility rating by a 5-point Likert scale**

1. Difficult to understand
2. Partly difficult to understand
3. Adequately understandable
4. Easy to understand
5. Very easy to understand

**Reliability rating by a 5-point Likert scale**

1. Not reliable: contains misinformation or major errors
2. Minimally reliable: some correct information, but key errors present
3. Moderately reliable: generally correct, but with some inaccuracies
4. Mostly reliable: accurate with minor omissions or uncertainties
5. Highly reliable: entirely accurate and trustworthy
